# Transmission risk of *Trypanosoma cruzi* through blood donors in a third-level care hospital

**DOI:** 10.1590/S1678-9946202567072

**Published:** 2025-10-13

**Authors:** Viridiana Gonzalez-Lopez, Carlos Alberto Zuñiga-Cruz, Sahara Vázquez-Ramírez, Emilio Rendon-Franco, Guiehdani Villalobos, Lucia Rangel-Gamboa, Fernando Martinez-Hernandez

**Affiliations:** 1 Hospital General “Dr. Manuel Gea González”, Tlalpan, Ciudad de México, Mexico; 2 Instituto Politécnico Nacional, Escuela Superior de Medicina, Sección de Estudios de Posgrado e Investigación, Casco de Santo Tomas, Ciudad de México, Mexico; 3 Universidad Autónoma Metropolitana, Departamento de Producción Agrícola y Animal, Coyoacán, Ciudad de México, Mexico

**Keywords:** T, cruzi, Blood transmission, Seroprevalence, Chagas disease, Chagatest

## Abstract

Blood transfusion is a major way of *Trypanosoma cruzi* transmission, a neglected parasite infection that produces Chagas disease (CD). CD has high morbidity and is potentially fatal in the chronic phase due to the parasite’s ability to invade the myocardium, colon, and esophagus. CD prevalence focuses on Latin America; however, its presence in non-endemic areas has been increasing in the last decade, particularly in North America and Europe, due to high migration rates of infected people. This study established the CD seroprevalence in a blood bank located in Mexico City. A total of 851 donor samples were analyzed by ELISA^n^ and Western blot^n^ (ELISA and WB using native antigens obtained from Mexican strains). Positive samples were analyzed by a commercial ELISA kit, named Chagatest (non-native antigens). Average donor age was 36.5 years, and most were men. Seroprevalence by ELISA^n^ and WB^n^ was 3.9% (33/851); whereas by Chagatest only six samples (0.7%) were reactive. Mexico City is not considered an endemic CD area; however, the high mobility of infected people increases the transmission risk in all receptor areas, particularly in blood banks, as seen in this study. Our findings showed a difference between native and non-native serological tests, and the need to maximize the identification of infected people to curb CD spread.

## INTRODUCTION

Chagas disease (CD) is caused by the protozoan parasite *Trypanosoma cruzi.* Affecting approximately 6 to 7 million people in Latin America, this infection is transmitted by vectors of the subfamily Triatominae. Other ways of acquiring the parasite include blood transfusion, organ transplantation, vertical transmission, and, less frequently, ingestion or laboratory accidents^1^.

The clinical presentation of CD comprises an acute and a chronic phase. Diagnosis in the chronic phase is based on serological tests like ELISA and Western blot, whereas diagnosis in the acute phase is based on parasitological methods, including PCR^1,2^. Usually, the acute phase is followed by a latency period. Patients in latency state are potential *T. cruzi* transmitters by blood transfusion^3^. Thus, transmission via blood transfusion has become the leading cause of *T. cruzi* infection in non-endemic areas and is the second path of acquiring the parasite (after vector transmission) in endemic zones^4^. In this scenario, searching for parasites in blood and organ donors is essential, especially in post-transplant patients who receive immunosuppressant treatment, as they could rapidly progress to systemic disease^5^. Reports of *T. cruzi* seroprevalence in Latin American blood banks range from 0.8% to over 20%^6^. In this context, this work evaluated CD seroprevalence in the blood bank of a third-level general hospital located in Mexico City (19°25’10”N 99°08’44”O), comparing the utility of native vs non-native antigens to identify seropositive Mexican patients.

## MATERIALS AND METHODS

### Ethics

This study was approved by the Ethics Committee of General Hospital “Dr. Manuel Gea Gonzalez” under Nº 12-10-2011. All procedures performed complied with the Helsinki Declaration of Human Rights and informed consent was previously obtained from all human adult participants.

### Serum samples

**Figure 1:**
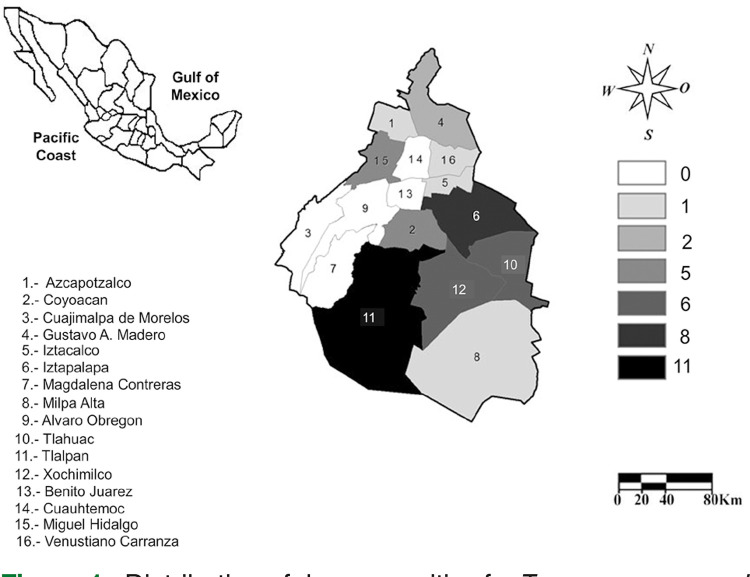
Distribution of donors positive for *Trypanosoma cruzi* antigen by ELISAn and WBn in Mexico City.

The sample comprised 851 blood donor serums which were collected between July and August 2012. The blood donors were people of both sexes, over 18 years old, clinically healthy, and without any declared chronic disease or risk factor for Chagas disease or other blood-borne infections. Blood donors included Mexico City residents and neighbors from Morelos and Mexico States (Figure 1). An epidemiological survey gathered basic characteristics of the study population, including age, sex, residence, and blood donation times.

### Parasite culture and antigen preparation

Epimastigote forms of native *T. cruzi* (DTU-I, MHOM/MX/1994/Ninoa) were cultured in liver infusion tryptose medium (LIT medium) supplemented with 10% fetal bovine serum previously inactivated at 56 °C for 30 min. Parasites were harvested at the exponential growth phase. For antigen preparation, parasites were collected by centrifugation at 1000g for 10 min at 4 °C, then washed and centrifuged twice in phosphate-buffered saline (PBS 1X, pH 7.2). The pellet was suspended in 5 mL of PBS, plus 5 mL of 10 mM Tris-HCl, pH 8.2, per gram of wet parasites. The following protease inhibitors were added: 5 mM ethylenediaminetetraacetic acid (EDTA); 1 mM phenylmethylsulfonyl fluoride (PMSF); 100 μM leupeptin; and 1 μM pepstatin. Parasites were sonicated using a Vibracell ultrasonic homogenizer (Sonics & Materials^®^, model VC130PB), and the mixture was centrifuged at 10,000 g for 30 min at 4 °C^7^. The supernatant (total extract) was recovered, and protein concentration was determined using a commercial Bradford kit (Bio-Rad Laboratories, CA, USA). The antigen was stored at −20 °C until use.

### Immunoassays using Mexican Ninoa strain as antigens

An enzyme-linked immunoenzymatic assay (ELISA) was standardized for detecting antibodies in serum^7^. EIA/RIA plates (Costar) were coated with 100 µL of solution containing 10 µg/mL total protein extract in 0.1M carbonate buffer, pH 9.6. Two hours after incubation at 37 °C, the plates were washed three times with PBS 1X/Tween 20 0.05% (PT buffer) and blocked for one hour at 37 °C with 1% bovine serum albumin in PBS. Donor serum was diluted 1:500 in PBS buffer at 100 µL/well, and plates were incubated for 2 h at 37 °C and then washed thrice as above. Next, 50 µL of peroxidase-conjugated anti-human IgG (Zymed Laboratory, Inc., San Francisco, CA, USA) diluted 1:7500 in PT buffer was added, and the plates were incubated for 2 h at 37 °C. After washing, the enzymatic reaction was induced with o-phenylenediamine and H_2_O_2_ as substrate. The reaction was stopped with 50 µL/well 2.5 N sulfuric acid. Absorbance values were determined at 490 nm (EPOCH, Biotek). Each test was performed in triplicate.

Following the methodology described by Sánchez *et al.* ^7^ and Padilla-Valdez *et al*.^8^, the positive and negative control sera obtained was used to calculate the cut-off value for the ELISA assay according to the equation: CO = [(X negative serum + (2.5 SD negative serum)], in which CO is the cut-off absorbance value, X= is the average absorbance of the negative serum, and SD= is the standard deviation of the absorbance values of negative serum. Samples were considered reactive if their absorbance values greater than the cut-off point and the positive and near-CO-positive serum samples were evaluated in triplicate. Negative sera showed constant optical density (OD) values in all assays^7,8^.

Results were expressed as the ELISA index (EI), in which EI = sample absorbance/cut-off absorbance. EI values > 1 were considered positive. The test showed a positive predictive value of 90% and a negative predictive value of 100%, with a kappa index of 0.96^7^.

**Figure 2:**
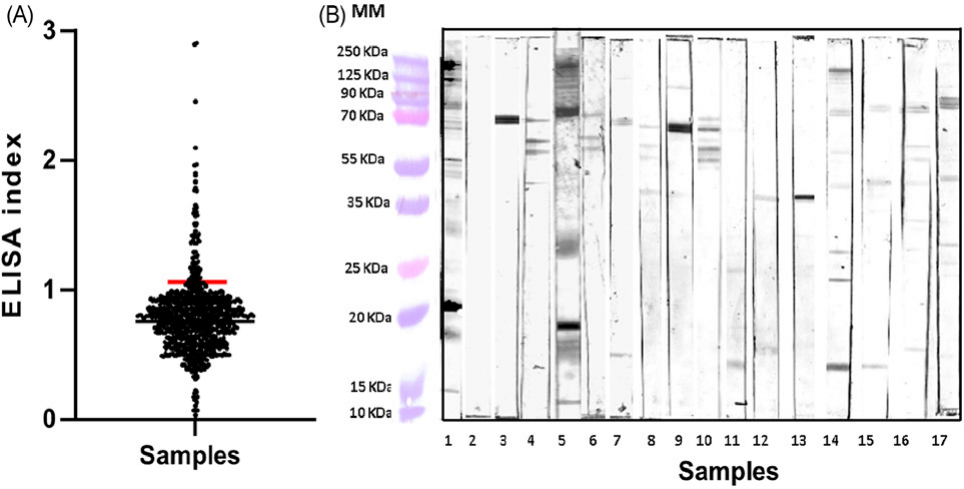
ELISA graphic and Western blot analysis: (A) ELISA plot of the 851 samples evaluated show the differences in the median seropositivity of antibodies (ELISA index [EI], in which EI = sample absorbance/cut-off absorbance) detected against the total protein extract of *T. cruzi*. Result values were considered positive when the ratio was greater than 1; (B) Representative Western blot analysis of the samples positive by ELISA test. MM = molecular markers; 1 = positive controls; 2 = negative control; 3-17 = Representative samples of the donors analyzed.

Western blot native (WB^n^) was performed as follows (Figure 2A). Proteins from the total parasite extract were separated by SDS-polyacrylamide gel electrophoresis (SDS-PAGE) and transferred to nitrocellulose membranes and cut into strips that were incubated individually overnight in PBS with 10% skim milk at 4 °C under constant agitation. Prior to this, the membranes of the nitrocellulose strips were incubated for 2 h at 37 °C with 1 mL of diluted human serum 1:250 in PBS with 10% skim milk. Each strip was washed thrice with PBS/0.1% Tween 20 and incubated with peroxidase-conjugated anti-human IgG (diluted 1:10,000) for 2 h at room temperature. After washing, the reaction was revealed with 0.5mg/mL diaminobenzidine in PBS with 0.02% H_2_O_2_. Reaction was interrupted with water. Positive and negative sera were included as controls in each experiment^7^.

A positive result was defined by the appearance of bands on the nitrocellulose membrane that corresponded to the pattern observed in the positive control. A negative result was recorded when no bands were visible, and an indeterminate result was defined by the presence of bands with a pattern differing from the positive control to exclude cross-reactivity with *Leishmania* ^7^.

### Commercial ELISA, Chagatest

Positive samples by ELISA^n^ and WB^n^ were evaluated by a commercial ELISA recombinant named Chagatest v.3.0 manufactured by Wiener Lab., Rosario, Argentina. According to the manufacturer, Chagatest uses recombinant antigens 1, 2, 13, 30, 36, and SAPA, obtained by DNA recombinant techniques from epimastigote and trypomastigote-specific proteins.

### Data analysis

Overall prevalence and descriptive statistics of the positive population were estimated. Agreement between ELISA^n^+WB^n^ and Chagastest was evaluated by Cohen’s Kappa value.

## RESULTS

### Serological test

A total of 851 serums were analyzed. Of these, 39 were positive by ELISA^n^ (Figure 2A), representing 4.6% (39/851), and 33 positive samples were confirmed by WB^n^(Figure 2B). WB^n^ results showed seventeen different antigenic proteins from blood donor sera. Proteins ranged from 10 to > 250 kDa. Most frequent proteins were 72 kDa in 58% of the sera; 32 kDa protein in 30% of the sera, and finally 50 kDa protein in 24%. Of the 33 samples positive by ELISA^n^ and WB^n^, only six were positive by ELISA Chagastest (Table 1). Thus, the agreement between WB^n^ + ELISA^n^ vs Chagastest calculated by Cohen’s Kappa value was 0.189, representing a slight agreement.

**Table 1:** Characteristic of the positive sera of blood donors with three techniques.

Nº	Sample	ELISA (Nativo)			Western blot (Nativo)		Chagatest
		O. D.	COV	O.D./COV	Bands number	Protein bands (kDa)	Results
1	10021	0.58	0.29	2.04	4	32, 55, 72, 95	-
2	10356	0.39	0.22	1.75	2	36, 50	-
3	11160	0.52	0.41	1.26	2	10, 70	-
4	11211	0.50	0.40	1.27	4	32, 55, 72, 120	-
5	11256	0.64	0.37	1.73	5	36, 40, 55,72, > 250 (1)	-
6	11860	1.63	0.35	4.72	5	28, 32, 45, 72, > 250 (1)	+
7	11863	0.50	0.35	1.45	2	23, 45	-
8	11878	0.70	0.36	1.97	6	25, 32, 50, 72, > 250 (2)	-
9	11887	0.81	0.43	1.91	1	72	-
10	11904	0.81	0.43	1.91	1	45	+
11	11911	0.74	0.45	1.63	1	50	-
12	11912	0.63	0.45	1.40	3	50, 70, 72,	-
13	11932	0.71	0.43	1.66	2	55, 72	-
14	11937	0.87	0.43	2.05	4	32, 50, 60, > 250 (1)	-
15	11939	1.32	0.43	3.09	3	32, 35, 50	-
16	11942	0.64	0.43	1.51	3	10, 70, 72	-
17	11954	0.56	0.43	1.30	2	72, > 250 (1)	-
18	11958	0.78	0.43	1.80	4	32, 36, 72, > 250 (1)	-
19	11964	0.71	0.41	1.75	2	25, 72	+
20	11966	0.61	0.41	1.49	2	50, > 250 (1)	+
21	11989	0.46	0.40	1.13	2	45, 72	-
22	11991	0.66	0.46	1.45	2	10, 45	-
23	11995	0.62	0.46	1.36	3	45, 70, 72	-
24	12010	0.77	0.52	1.50	1	> 250 (1)	-
25	12012	0.65	0.52	1.25	1	40	-
26	12040	0.80	0.55	1.47	4	40, 42, 72, > 250(1)	-
27	12060	0.58	0.41	1.40	4	32, 50, 72, > 250 (1)	-
28	12120	0.65	0.40	1.65	1	> 250	+
29	12123	0.70	0.40	1.76	3	32, 60, 72	-
30	12174	0.47	0.28	1.66	2	36, 55	-
31	12177	0.62	0.35	1.78	3	45, 72, > 250 (1)	-
32	12197	0.64	0.41	1.56	3	55, 60, 72	+
33	12287	0.39	0.29	1.36	3	25, 32, 95	-

OD = optical density; COV = optical density cut-off value; ELISA index O.D./COV.

### Epidemiological data

Among the analyzed sera, 84% correspond to men and 16% to women, with an approximate ratio of 5:1, respectively. Average donor’s age was 36.5, ranging from 18 to 59 years of age.

Overall, all positive samples corresponded to people between 31-40 years of age (26.8%), followed by 21 to 30 years old (25%). Donor’s place of residence included thirteen geographic areas from the 16 city halls that make up Mexico City (Figure 1). Most donors resided in Tlalpan (19.6%) and Iztapalapa (14.3%). Locations outside of Mexico City were Morelos and Mexico State with 3 and 5 donors, respectively.

As for the number of donations per participant, 30.3% were first-time donors (10/33), followed by 45.5% (15/33) second-time donors. However, some individuals have donated three (3/33), four (2/33), five (6/33), six (2/33), and even eleven times (1/33) before.

## DISCUSSION

Chagas disease is a critical health problem in the American continent. Previously considered an endemic issue, nowadays the spread of *T. cruzi* infection has grown significantly in areas where transmission had previously been absent or very low due to increased travel and immigration^9^. One of the crucial factors in disease transmission in non-endemic areas is blood transfusion, with studies showing this point in areas such as North America^9^, Europe^10,^ Asia^11^, and Oceania^12^.

In Mexico, data on blood transfusion-transmitted CD cases are scarce. Epidemiology studies reported national seroprevalences varying from 0.46% to 1.6% by commercial methods, but other studies found seropositive values between 0.17% and 20%. This reported variation is first related to geographic regions^13^. Our results indicate a *T. cruzi* seroprevalence of 3.9%. Previous studies did not consider Mexico City as an endemic area for CD. However, our results can be explained by the internal migration phenomena occurring in the last century, as Mexico City receives people from all states, including migrants from Chagas-endemic rural areas. The Mexican National Institute of Statistics Geography and Informatics (INEGI) reported between 2015 and 2020 that the number of internal migrants established in Mexico City was 308,686 people. Most of which were originally from CD endemic areas, such as Veracruz, Puebla, Oaxaca, Guerrero, Morelos, and Mexico states^2,3^. As observed in our data, all donors are evenly distributed throughout the city; thus, the risk of *T. cruzi* transmission by transfusion is indeed possible in Mexico City, even if it is considered a non-endemic region^14^. Additionally, the official regulation for blood banks in Mexico (NOM-253-SSA1-2012) has established the diagnosis of *T. cruzi* in blood transfusions as a mandatory measure only since 2012, with the only specification that the commercial methods to be used report a sensitivity of 99.5% and a specificity of 99%. However, the method used to determine seroprevalences may present discrepant results as shown by Ballinas-Verdugo *et al*.^15^, who determined the *T. cruzi* seroprevalence in Mexico City donors by ELISA-INC9 (which used native antigen obtained from Mexican strain INC9) and Chagatest and found seroprevalence results of 4.3% and 0.7%, respectively. Similarly, Padilla-Valdez *et al*.^8^ also observed clear differences between commercial tests and ELISA with native antigens, showing a 0% correlation between both techniques.

This highlights the difficulties in diagnosing Chagas. Thus, the real prevalence of *T. cruzi* in Mexican blood banks could be underestimated when determined by commercial methods that use antigens obtained from non-native South American strains^16^.

Differences in immunological response, level of virulence, and antigen production in the *T. cruzi* Mexican strain have been investigated. For example, Ninoa and INC5 strains differentially infect and modulate MHC-II, toll-like receptors and cytokine production in dendritic cells (BALB/c mice) compared with the CL-Brenner strain (Brazil)^17^. This heterogenous behavior among strains could explain the diversity of antigens recognized by ELISA and WB, as a result of the existing differences in *T. cruzi* populations and the geographic areas where CD was acquired. The most frequent proteins found by WB^n^ were 32, 50, and 72 kDa. However, their recognition did not exceed 30%, suggesting a high complexity between the immune response, human genetics, and the diversity of *T. cruzi* strains.

Moreover, this work highlights the importance of using local strain to obtain antigens for ELISA and WB or other techniques that show high sensitivity like TESA^18^, since most positive cases were not detected by Chagastest. Commercial ELISA is effective in detecting high titers of anti-*T. cruzi* antibodies but questionable in people with low titers, possibly associated with the used antigens which were obtained from laboratory strain originally from other countries and corresponded to a *T. cruzi* DTUs not common in Mexico. This hallmark was previously reported by Sanchez *et al*.^7^. DTU-I is the predominant genotype in Mexican patients, mostly associated with sylvatic or peridomestic niches, also prevalent in Central America and Sout America northern countries (Colombia, Venezuela Guayana, and Suriname). Below the equator, DTU-II and V have the highest presence^19^. Hence why we highlight the importance of mandatory detection of *T. cruzi* in Mexico City blood banks, considering that an unsafe blood supply is costly in both human and economic terms.

Regarding donor antecedents, during the interview before blood donation, the data indicating donor interaction with the vector or data suggesting parasite infection are based on questions such as: 1. Do you know people with a positive diagnosis of *T. cruzi*?; 2. Do you have a family member (mother) positive for Chagas?; 3. Do you live with kissing bugs (triatomines) in your home or report being bitten by them? Considering recent data, we propose that possible donors also must be interviewed for the presence of prolonged dermatological lesions and recent visits to endemic states, especially rural areas or countryside, since, as reported by Rangel-Gamboa *et al.* ^20^ *,* patients infected with *T. cruzi* DTU-II in Mexico showed prolonged cutaneous lesions with delayed seroconversion. Thus, constant surveillance of *T. cruzi* in blood banks provides data that contributes to monitoring CD, but more importantly, to stopping this silent transmission route.

Finally, we recognize that our study has some limitations like the use of the same antigen in the native ELISA and WB, the results of monitoring or re-evaluation of those samples close to the cut-off points (as this is a cross-sectional study), and the comparison with other highly sensitive and specific techniques such as those based on trypomastigote excreted/secreted antigen (TESA)^18^ to determine more precisely the importance of Chagas disease in blood banks. However, our study is one of the few that analyzes a large number of samples and compare specific techniques with a rapid diagnostic test (RDT).

## CONCLUSIONS

Our results indicate a *T. cruzi* seroprevalence of 3.9%, confirmed by two techniques. To explain this high prevalence, we considered two events: first, the human migration from Chagas endemic country sites to Mexico City; second, the clear difference in diagnosis results observed between commercial kits versus techniques that use native antigens. Finally, WB^n^ showed a high frequency of recognition of proteins 72, 32, and 50 kDa. Despite a heterogeneous recognition of antigens, none showed 100% recognition.

## Data Availability

The complete anonymized dataset supporting the findings of this study is included within the article itself.
